# Osteoporosis and sarcopenia are common and insufficiently diagnosed among chronic pancreatitis patients

**DOI:** 10.1186/s12876-023-02756-w

**Published:** 2023-04-13

**Authors:** Mikael Parhiala, Mika Ukkonen, Juhani Sand, Johanna Laukkarinen

**Affiliations:** 1 Faculty of Medicine and Health Technology, Tampere University, Tampere, Finland; 2 Department of Gastroenterology and Alimentary Tract Surgery, Tampere University Hospital, Elämänaukio Kuntokatu 2, 33520 Tampere, Finland

**Keywords:** Vitamin deficiency, Pancreatic insufficiency, Exocrine insufficiency, Alcohol, Pancreatic enzyme replacement therapy, Bone mineral density, Psoas muscle area

## Abstract

**Purpose:**

Chronic pancreatitis (CP) leads to diabetes and pancreatic exocrine insufficiency (PEI). PEI may lead to maldigestion and malnutrition, which may cause fat-soluble vitamin deficiency, sarcopenia and abnormal bone density. We aim to study the prevalence of osteoporosis, sarcopenia and vitamin deficiency among CP patients.

**Methods:**

Long-term (4–5 years) follow-up was implemented on CP patients. We recorded CP duration, BMI, smoking, alcohol consumption and medication. We determined the serum values for A, D and E vitamins, albumin, creatinine, haemoglobin, calcium and magnesium. Bone density measurement was taken from the proximal femur and lumbar spine. CT/MRI scans were used to measure for psoas muscle area.

**Results:**

A total of 33 patients (median age 62 [39–81] years, 61% male) were included. None of these patients had earlier diagnosis of osteopathy, and none of them had known vitamin deficiency or were sarcopenic. Nineteen patients (57%) had pancreatic exocrine insufficiency and of these seven patients (37%) had no pancreatic enzyme replacement therapy (PERT) and one (5%) had inadequate enzyme therapy. During the study, osteoporosis was diagnosed in 20% and possible sarcopenia in 48% of patients. PEI and inadequate PERT was associated with low E vitamin levels (75% vs. 0%, *p* = 0.012), higher risk of osteoporosis (43% vs. 5.6%, *p* = 0.013) and sarcopenia (80% vs. 36%, *p* = 0.044).

**Conclusion:**

This study demonstrates that chronic pancreatitis is associated with osteoporosis, sarcopenia and vitamin deficiency. If untreated, pancreatic exocrine insufficiency is associated with increased risk of these outcomes. This highlights the importance of identifying and treating PEI in CP patients.

## Introduction

Chronic pancreatitis (CP) manifests as a persistent or intermittent inflammation of the pancreas, which may over time cause morphological changes to the pancreatic tissue. This can lead to permanent pancreatic exocrine (PEI) and endocrine insufficiency. The exocrine pancreas has an essential role in the digestive system secreting digestive enzymes (e.g., lipase, trypsin and amylase) in a bicarbonate solution which break down fats, proteins and carbohydrates. Exocrine insufficiency can lead to steatorrhoea, maldigestion and malnutrition. Overall exocrine insufficiency may impose patients to fat-soluble vitamin deficiency, sarcopenia and osteoporosis [1–4].

Osteoporosis is defined as a skeletal disease with low bone strength leading to an increased risk of fractures, while sarcopenia is defined as loss of muscle mass and impaired physical performance [5, 6]. Diagnosis of osteoporosis is based on bone mineral density (BMD), typically measured from the femoral neck or the lumbar bone [7]. Sarcopenia and osteoporosis have both been found to be associated with higher mortality, lower quality of life (QoL) and increased risk of hospitalizations. There is recent evidence that CP patients could have sarcopenia with an incidence of 17–62% with exocrine insufficiency and pancreatic fibrosis being a suspected risk factor [8, 9]. There is also evidence that almost 25% of CP patients have osteoporosis [10] and are at risk for vitamin deficiency [11]. Since all these conditions are interlinked in that they are caused by probable malnutrition, maldigestion, possible alcohol consumption and chronic inflammation our aim was to determine how possible sarcopenia, osteoporosis and vitamin deficiency were diagnosed and treated in CP patients. The nutritional status and osteoporosis and possible sarcopenia of CP patients in Finland have not been studied.

## Methods

**Fig. 1:**
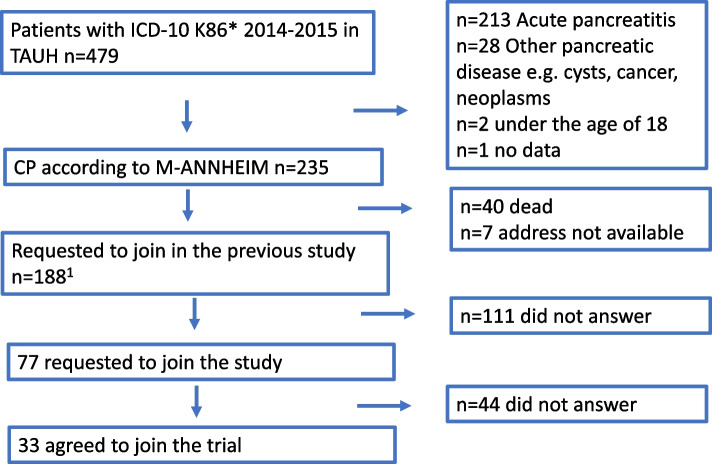
Flowchart of patient selection in the study. We selected all chronic pancreatitis (CP) patients from 2014 and 2015 consecutively. All patients in the study had definitive diagnostic characteristics according to M-ANHHEIM. *1) Parhiala et al. 2020 Pancreatology*

Consecutive patients with CP at Tampere University Hospital, Finland between 1 January 2014 and 31 December 2015 were included. CP was defined according to the definitive diagnostic criteria of CP according to the M-ANNHEIM criteria in a study illustrated in Fig. 1 [12, 13]. The definitive diagnostic criteria include one or more of the following: Enlargement of the main pancreatic duct, (moderate or marked ductal pancreatic ductal lesions according to the Cambridge classification), pancreatic calcification, pancreatic exocrine insufficiency with pancreatic steatorrhoea clearly reduced by pancreatic enzyme replacement therapy (PERT) or a typical histological specimen of the pancreas.

Data regarding testosterone, albumin and vitamin D, E and A levels were measured in 2019. We also elicited information on possible fractures, medication, vitamin supplements and menopause. We measured the participants’ weight and height and calculated the body mass index (BMI) kg/m^2^. BMI ≥ 25 kg/m^2^ was considered overweight and BMI ≥ 30 kg/m^2^ was considered obese [14]. The patients provided blood and stool samples and a bone density measurement was taken. A faecal elastase-1(FE-1) level less than 100 μg was considered clinically relevant. An adequate amount of PERT was defined as 25–50 000 IU of pancreatic lipase per meal according to the 2020 ESPEN guidelines [15, 16].

### Bone density measurement

Bone density measurement or dual-energy x-ray absorptiometry (DXA) was done at both femur neck and lumbar spine in 2019. The Lunar iDXA (GE Medical Systems, Milwaukee, Wisconsin, USA) with enCORE v16 software was used for all bone density measurements. The measurements were based on the World Health Organization definition of a T score of -2.5 or less. Osteopenia defined as T score between -1 and -2.5. In patients under the age of 50 years we used a Z score instead of a T score according to the International Society for Clinical Densitometry guidelines. A Z score of -2.0 or under is “below the expected range of age” [17, 18].

### Sarcopenia measurement

Psoas muscle area (PMA) was identified by CT or MRI scans gathered retrospectively within one year from the time when the DXA measurement was taken between the years 2018 and 2020 [19]. We calculated the mean area (mm^2^) of both left and right psoas muscles from the middle of the third lumbar vertebra, so that both transverse processes were visible. The psoas muscle area was precisely drawn by the same clinician and area calculated (mm^2^) using the Sectra Workstation version 23.1 (Sectra AB, Linköping, Sweden). This method had been previously tested and described to be applicable by a single clinician in a routine clinical setting [20]. To define possible sarcopenia we used a PMA of under 800 mm^2^ for males and under 550 mm^2^ for females as a cut-off based on an earlier Finnish study [21].

All the data except for aetiology and PMA from CT/MRI scans were gathered prospectively.

Data are presented as medians (with min–max) if variables were not normally distributed or as averages (standard deviation) if variables were normally distributed. The statistical analysis was calculated using Pearson’s Chi-Square or in continuous values, the Mann–Whitney U test (not normal distribution) or Student’s t-test (normal distribution) was used. For correlation calculation we used the Pearson’s Correlation Coefficient test (r). The odds ratio (OR) was calculated using binary logistic regression and are presented with a 95% confidence interval (CI 95%) Statistical analyses were done with IBM SPSS v28 (IBM Corp, Armonk, New York, USA). A *p*-value of under 0.05 was considered statistically significant.

## Results

A total of 33 patients (median age 62 years [range 39–81] years) were included, with a median disease duration of six (range 4–27) years. Aetiology for CP was alcohol related in 49% (*n* = 16) of the cases. Thirty-six percent of patients (*n* = 12) were active smokers and 64% (*n* = 21) had a smoking history with a median of 30 (4–60) pack-years of smoking. Median BMI was 26.9 (18.8–38) kg/m^2^, 66% of the patients were overweight and 24% obese. Forty-seven percent of patients with PEI were overweight and 11% obese, compared to 93% and 43% of those without PEI. Sixty-four percent of patients (*n* = 21) had a daily vitamin D substitute median 20 (10–75) μg and 15% (*n* = 5) had low levels of vitamin D. Fifty-two percent of patients (*n* = 17) had undergone interventions due to CP related complications. Surgery was performed on seven patients: including three pancreatic resections and four due to pseudocystal or pancreatic fistula complications. Pancreatic surgery was performed a median of four years [2–5] before the study. Endoscopic procedures were performed on eleven (33%) patients. Nineteen patients (57%) had PEI (FE-1 levels under 100) and out these seven patients (37%) had no PERT and one (5%) had inadequate PERT consumption. Patient characteristics are presented in Table 1.

**Table 1:** Demographic of chronic pancreatitis patients in the study

Chronic pancreatitis patient	Male (*n* = 20)	Female (*n* = 13)	Total (*n* = 33)
Age median years (range)	61 (44–81) years	62 (39–79) years	62 (39–81) years
Time after diagnosis (range)	6.5 (4–16) years	6 (4–27) years	6 (4–27) years
Alcohol aetiology	55% (11)	38% (5)	49% (16)
Smoking	75% (15)	46% (6)	64% (21)
Elastase under 200	75% (15)	77% (10)	75% (25)
Elastase under 100	55% (11)	62% (8)	57% (19)
PERT	65% (13)	77% (10)	70% (23)
Inadequate PERT and PEI	55% (6)	25% (2)	42% (8)
Diabetes	95% (19)	69% (9)	75% (25)
BMI	27 (19–34)	28 (21–38)	28 (19–38)
Menopause	-	77% (10)	77% (females only)
Low Testosterone	10% (2)	-	10% (males only)
Osteoporosis	20% (4)	23% (3)	21% (7)
Osteopenia	25% (5)	31% (4)	24% (8)
Normal Bone Density	55% (11)	46% (6)	55% (18)
Possible sarcopenia	50% (7/14)	43% (3/7)	48% (10/21)
Endoscopic procedure	30% (6)	38% (5)	33% (11)
Pancreatic surgery	25% (5)	2 (15%)	21% (7)

*BMI*, Body Mass Index *PES*, Pancreatic enzyme supplements, *PEI* Pancreatic exocrine insufficiency, *PERT* Pancreatic Enzyme Replacement Therapy

### Osteoporosis, osteopenia and osteoporotic fractures

None of the CP patients were known to have had osteoporosis, osteopenia or osteoporotic fractures prior to the study. Forty-five percent of males and 58% of females had abnormal bone density. Osteoporosis was diagnosed in 20% (*n* = 7) and osteopenia in 23% (*n* = 8) of patients. Twenty percent of the males (median age 62 [44–69] years) had osteoporosis and 23% of the females (median age 62 [39–79] years) had osteoporosis, while 45% of males and 58% of females had abnormal bone density. Patient characteristics are presented in Table 2.

**Table 2:** Demographic chronic pancreatitis patients with osteoporosis. None of the CP patients had previously known osteoporosis, osteopenia or osteoporotic fractures before the study

	***Osteoporotic bone density* < *-2.5 Z-score* (***n*** = 7)**	***Bone density* > *-2.5 Z-score* (***n* **= 26)**	***p value***
Age median (range)	**64 (44–79)**	**60 (39–81)**	*0.27*
Female/male ratio	**43%/57%**	**38%/62%**	*0.83*
Time after diagnosis (years)	**7 (5–15)**	**6 (4–27)**	*0.32*
Elastase-1 (µg/g) med (range)	**20 (15–387)**	**53 (15–500)**	*0.81*
Elastase under 200	**57% (4)**	**81% (25)**	*0.20*
Elastase under 100	**57% (4)**	**77% (20)**	*0.98*
PERT	**43% (3)**	**77% (20)**	*0.08*
Low elastase and no PES	**43% (3)**	**15% (4)**	*0.28*
Possible sarcopenia	**67% (2/3)**	**44% (8/18)**	*0.48*
Alcohol aetiology	**71% (5)**	**42% (11)**	*0.17*
Alcohol abstinence	**29% (2)**	**48% (12)**	*0.17*
Current smoking	**57% (4)**	**27% (7)**	*0.20*
Smoking	**86% (6)**	**58% (15)**	*0.32*
Idiopathic pancreatitis	**0%**	**19% (5)**	*0.21*
BMI	**28.3 (19.9–29.1)**	**27.3 (18.8–38)**	*0.62*
Overweight	**57% (4)**	**69% (18)**	0.55
Obese	**0%**	**31% (8)**	0.09
Haemoglobin (g/l)	**145 (118–167)**	**138 (88–162)**	*0.11*
Creatinine (µmol/l)	**75 (66–91)**	**75 (58–131)**	*0.61*
Glomerular filtration rate (GFR)	**89 (53–101)**	**89 (50–116)**	*0.11*
Albumin (36–45) (g/l)	**37 (32–42)**	**37 (24–41)**	*0.18*
Fasting blood sugar (4–6.1) (mmol/mol)	**6.4 (5.5.-13.9)**	**6.9 (5.8–16)**	*0.28*
HbA1c (20–42) (mmol/mol)	**41 (36–69)**	**46 (35–74)**	*0.13*
Triglycerides (< 1,7) (mmol/l)	**0.89 (0.80–2.04)**	**1.33 (0.59–14.23)**	*0.29*
Vitamin D 1.25 levels (50–375) (nmol/l)	**54 (33–70)**	**65 (33–108)**	*0.14*
Calcium (2,15–2,51) (mmol/l)	**2.31 (2.20–2.37)**	**2.34 (2.27–2.62)**	*0.12*
Magnesium (0,71–0,94) (mmol/l)	**0.84 (0.81–0.95)**	**0.80 (0.57–0.97)**	*0.18*
Vitamin A levels (0,7–4,2) (μmol/l)	**1.6 (0.8–3.2)**	**1.9 (1–3.3)**	*0.29*
Vitamin E-levels (12–37) (μmol/l)	**19 (8–40)**	**24.5 (10–70)**	*0.14*
Low testosterone in males	**0%**	**12.% (2)**	
Menopause in females	**100%**	**0.0%**	
Vitamin D supplementation	**71% (5)**	**65% (16)**	*0.63*
Cortisone medication	**14% (1)**	**0%**	
Endoscopic procedures	**2 (29%)**	**9 (35%)**	0.76
Surgery	**0 (0%)**	**7 (27%)**	0.12

*BMI* Body Mass Index, *PES* Pancreatic enzyme supplements, *PERT* Pancreatic Enzyme Replacement Therapy

Alcohol consumption after diagnosis of CP was associated with abnormal bone density: 67% in patients with alcohol consumption vs. 29% in patients without alcohol consumption, *p* = 0.035 but there was not statistical difference alcohol consumption and osteoporosis: 33% with alcohol consumption vs. 12% without alcohol consumption, *p* = 0.141. Smoking (*p* = 0.171), older age (*p* = 0.268), female gender (*p* = 0.833), BMI (*p* = 0.620) and low testosterone (*p* = 0.456) were not associated with higher risk of osteoporosis.

Median BMI of the patients with osteoporosis was 28.3 (19.9–29.1) and 27.3 (18.8–38) among non-osteoporotic patients (p = 0.62). None of the obese patients (BMI > 30) had osteoporosis (*p* = 0.092; not significant). Low testosterone levels in males were detected in two CP patients but neither had osteoporosis. The female osteoporotic patients were all post-menopausal and one was taking long lasting cortisone medication. None of the patients who had pancreatic surgery had osteoporosis, this did not reach a statistical difference (*p* = 0.122).

The patients with PEI and no PERT had more osteoporosis than did CP patients with PERT and PEI 43% vs 5.6% (OR 2.3 CI 95%: 0.8–6.9; *p* = 0.013).

### Psoas muscle area

**Fig. 2:**
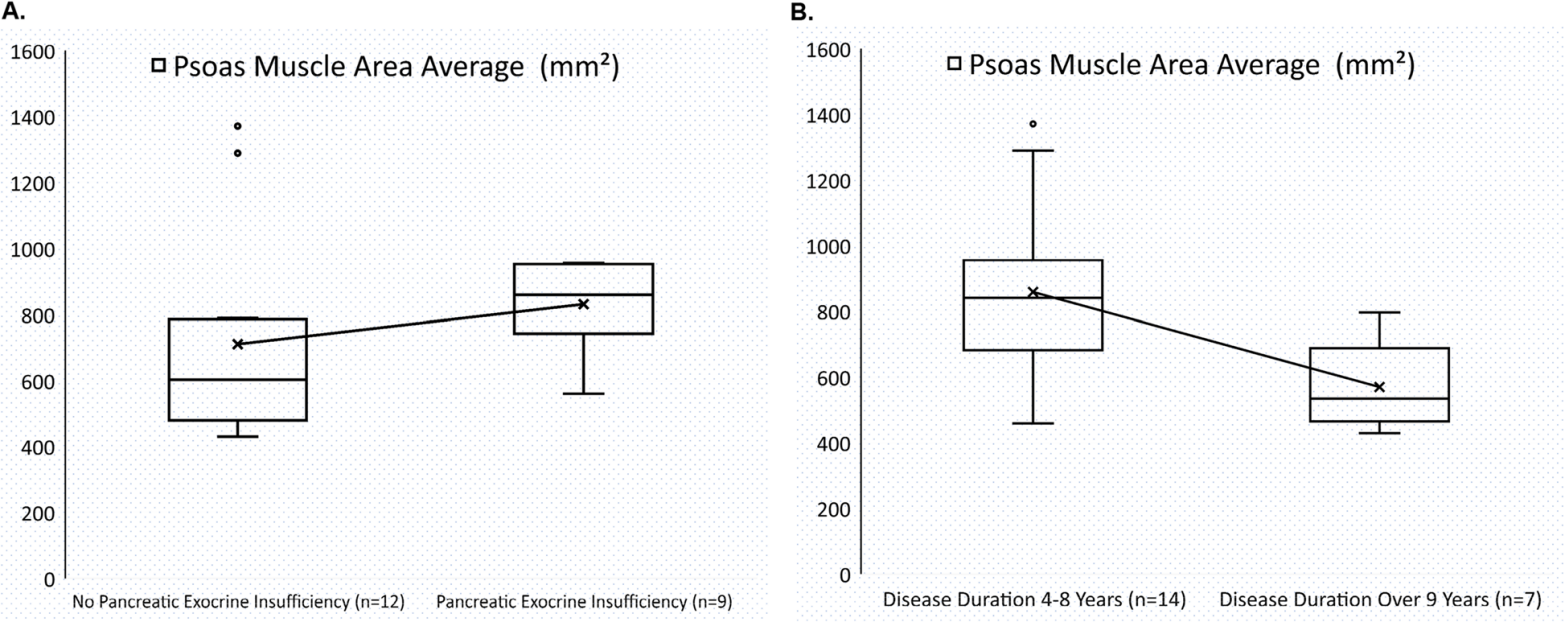
Boxplot of psoas muscle area (PMA). **A** Demonstrating a trend towards lower PMA with pancreatic exocrine insufficiency (PEI). **B** Demonstrates a lower PMA with disease duration of over 9 years despite there being no age difference

Psoas muscle area (PMA) was measured in 21 patients. The median PMA for females was 561 (430–956) mm^2^ and for males 809 (467–1371) mm^2^. Possible sarcopenic PMA levels were registered in 48% (*n* = 10) of the patients. PEI was found in 80% of the sarcopenic group vs. 36% in non-sarcopenic patients (OR 7.0 (95% CI:0.97–50.6); *p* = 0.044). The trend between PEI and low PMA is present in Fig. 2A. There was no difference in patients with PEI and no PERT in the possibly sarcopenic and non-sarcopenic group (*p* = 0.157). Longer disease duration was correlated with lower PMA (r -0.434, *p* = 0.049), age did not correlate with lower PMA (r 0.263, *p* = 0.249). Patients with possible sarcopenia had median nine [4–27] years’ history of CP, compared to five [4–8] of those without sarcopenia, *p* = 0.002. The association between disease duration and PMA is illustrated in Fig. 2B.

### PEI and vitamin deficiency

PEI was found in 57% (*n* = 19) of the patients and was associated with alcohol consumption after diagnosis of CP 80% vs. 35%, *p* = 0.011. Smoking was not associated with PEI (*p* = 0.947). Of the PEI group 21% (*n* = 4) had low levels of vitamins D and E.

In the non-PEI group all patients had normal levels of vitamin E and one1 had a low level of vitamin D. All low vitamin E levels were found in the PEI group (*p* = 0.067). Three (75%) were found in CP patients with inadequate PERT. CP patients with PERT had less vitamin E deficiency than CP patients without PERT (OR 14.4 (95% CI:1.2–169); *p* = 0.01).

They also had lower levels of vitamins D and A, but the difference was not statistically significant. No low levels of vitamin A were measured. Of all participants15% (*n* = 5) had low vitamin D levels. Most of patients with PEI 74% had supplementary vitamin-D (74%) and half of the non-PEI group had supplementary vitamin-D (*p* = 0.162).

## Discussion

CP patients carry a high risk for osteoporosis and for osteoporotic fractures [22, 23]. It was unknown how this is addressed in daily clinical practice. In our study, nearly half of the CP patients had abnormal bone density while none of them had been diagnosed with osteoporosis or osteopenia. CP patients with PEI and no enzyme substitute had more osteoporosis than CP patients with PEI and enzyme substitute. CP patients with PEI and long disease duration were found to have an increased risk for sarcopenia.

The United European Gastroenterology 2018 guidelines have previously recommended DXA and serum vitamin D (25-hydroxyvitamin D3) measurement in CP patients [24]. Our findings support these recommendations. As we demonstrated, osteoporosis is common among CP patients, and none of our study patients had known osteoporosis when they were enrolled for this trial. The Finnish guidelines for screening for osteoporosis focus on high fracture risk patients or patients who have already sustained fractures [25]. Osteoporosis has multiple risk factors, the most common being age, menopause, glucocorticosteroids, low peak bone mass and immobilisation with multiple risk factors related to CP such as diabetes, high alcohol intake, smoking and malabsorption [26–28]. The authors recommend screening high-risk patients, also those with CP.

More detailed follow-up might serve to reduce the risk not only of osteoporosis and osteoporotic fractures, but also of vitamin deficiency and sarcopenia. CP patients have a higher mortality rate than to controls with known risk factors such as diabetes and smoking, but it should also be taken into account that PEI may be an independent risk factor for low survival and should be treated appropriately [29–31].

The cause of sarcopenia and osteoporosis in CP is multifactorial and includes nutritional components: maldigestion of fats and fatty vitamins, chronic inflammation, alcohol and smoking [32]. In our study half of the patients had low PMA indication of possible sarcopenia and this was associated with PEI and longer disease duration. Sarcopenia has been associated with lower survival and increased hospitalisation in CP patients [33]. Even though no evidence has so far been presented that sarcopenia is related to a higher complication rate in CP patients, a connection to failure to overcome complications related to surgery has been reported [34–36]. Sarcopenia in CP could be due to malnutrition and assessment and prevention of this needs to a focal point in treatment of CP [37].

Since low physical activity has been linked to both sarcopenia and osteoporosis there is no studies investigating the effect of physical activity on CP patients [38].

None of the patients who underwent pancreatic surgery for CP had osteoporosis. There is no literature that we know of looking into bone density in CP patients after surgery. This needs further exploration.

We found it concerning that nearly 40% of the patients with PEI had no pancreatic enzyme substitute. This could be due to lack of follow-up and poor compliance in CP patients [39]. More patient education on PEI should be provided. Exocrine insufficiency can be diagnosed via FE-1, faecal fat collection or C13 mixed triglyceride and is treated with a pancreatic enzyme replacement therapy PERT taken at every meal [4]. An adequate number of PERT is needed to treat PEI. The United European Gastroenterology guidelines for the treatment of CP recommend 40–50 000 IU lipase for meals and 20–25 000 IU for smaller meals [40]. In our study 12% of CP patients had low levels of Vitamin E, a risk factor being PEI with inadequate PERT consumption. Vitamin E is a fat-soluble antioxidant, low levels being extremely rare in normal population [41, 42]. Vitamin E deficiency may cause neurological disorders [43]. An earlier study found that low levels of Vitamin E were common (10%) in CP patients with PEI, especially if no PERT was used [42]. Vitamin E levels should be measured and supplementary vitamin-E given with a low threshold, especially to patients with PEI. We did not measure any low levels of Vitamin A. Low levels of vitamin A have only been reported in CP patients with PEI and without PERT [44].

In Finland the prevalence of vitamin D deficiency in adults is 21–26%, which is actually higher than in our selected population (15%). Moreover, in our study CP patients had a higher rate of vitamin D supplementation than the average Finnish population 74% vs. 57% [45]. This could be due to selection bias in our study population.

We found that patients who continued to consume alcohol after their diagnosis of CP had more abnormal bone density and PERT. Patient education concerning CP is insufficient and better tools for reaching these patients are needed [46]. In a 2021 retrospective study Srivoleti et al. found that less than half of the patients followed recommendations regarding lifestyle changes for CP and that patients treated by pancreatologists were more likely to abstain from alcohol [47].

In a recent international survey 75% of pancreatologists prescribed PERT in clinically evident steatorrhea while only 20% of clinicians routinely checked for PEI and conducted nutritional tests during follow-ups [48]. This may suggest a need for guidelines and a change of mindset in the treatment of CP. In our study two out of five of CP patients with PEI did not receive sufficient treatment. In our study patients without proper PERT, with osteoporosis or vitamin deficiency were contacted and advised to contact their physician.

It must be stated that FE-1 is considered to be unspecific for excluding mild or moderate PEI and consensus is lacking regarding the ideal cut-off value [40]. The most used cut-off value is 200 faecal μg per one gram of faeces. In this study we used a cut-off value of 100 μg FE-1 due to it being more specific and having the same sensitivity as the previously used 200 μg [16].

A strength of the study is its prospectively gathered data on bone density, medication and laboratory test in this somewhat hard-to-reach patient group. Among the limitations is that we were unable to measure vitamin K levels, which might have had an impact on bone metabolism. Sarcopenia is defined as loss of muscle mass and function. In this study we were able to assess only muscle mass. Furthermore, we only measured muscle mass of psoas muscle. Whether this represents overall sarcopenia remains beyond the scope of this study. We used imaging CT/MRI imaging to asses possible sarcopenia which still remains the primary modality for assessment of sarcopenia due to availability and ease of use [49] and it is still considered a practical method of assessing sarcopenia [50].

Next, we were able to assess psoas muscle in about two thirds of patients, which may have caused some patient selection bias. A low number of patients recruited in our study illustrates problems in performing studies in selected group of patients. While this study was conducted over several years in high volume centre, only a small number of patients took part in long-term follow-up. Chronic pancreatitis is relatively rare condition and attending patients have high drop-out rate. In our earlier study the drop-out rate was 60%, while it was 57% in this study. High drop-out rate may cause some patient selection bias, as those motivated for trials may be also more motivated to change their life habits, ea. alcohol and tobacco use. Nevertheless, we emphasize that all those with chronic pancreatitis may require more holistic approach in future. Similar problems with low patient count are likely to arise in multicentre trials. Among the strengths was that we were able to conduct this population-based trial including all those with chronic pancreatitis living within a hospital district of a high-volume centre with comprehensive data available on all attending patients.

In conclusion, osteoporosis, osteopenia, sarcopenia and vitamin deficiencies are common in CP patients. In our study, all the osteoporosis was previously undiagnosed. More care should be taken in the basic treatment of PEI in this patient group. We concede that there is a need for improvement in the treatment of pancreatic exocrine insufficiency in CP patients. This study opens opportunities for interventional prospective studies with interventions for clinicians to gain a better understanding of how to treat CP and PEI and to prevent or delay the progression of osteoporosis and sarcopenia in CP patients.

## Data Availability

The data regarding this study are available on request from the corresponding author. The data are not publicly available due to their containing information that could compromise the privacy of research participants.

## Abbreviations

BMD: Bone Mineral Density
BMI: Body Mass Index
CP: Chronic Pancreatitis
DXA: Dual-energy X-ray Absorptiometry
FE-1: Faecal Elastase-1
PEI: Pancreatic Exocrine Insufficiency
PERT: Pancreatic Enzyme Replacement Therapy
PMA: Psoas Muscle Area
QoL: Quality of Life
